# Marker-Less Navigation System for Anterior Cruciate Ligament Reconstruction with 3D Femoral Analysis and Arthroscopic Guidance

**DOI:** 10.3390/bioengineering12050464

**Published:** 2025-04-27

**Authors:** Shuo Wang, Weili Shi, Shuai Yang, Jiahao Cui, Qinwei Guo

**Affiliations:** 1 Department of Engineering Physics, Key Laboratory of Particle and Radiation Imaging, Ministry of Education, Tsinghua University, Beijing 100084, China; shuo-wan19@mails.tsinghua.edu.cn; 2 Department of Sports Medicine, Beijing Key Laboratory of Sports Injuries, Peking University Third Hospital, Institute of Sports Medicine of Peking University, Haidian District, Beijing 100191, China; shiweilixmu@126.com (W.S.); yangshuaibd@pku.edu.cn (S.Y.); 3 State Key Laboratory of Virtual Reality Technology and Systems, Beihang University, Beijing 100191, China; cuijh@buaa.edu.cn

**Keywords:** anterior cruciate ligament reconstruction, computer-assisted surgery, arthroscopic navigation, femoral tunnel positioning

## Abstract

Accurate femoral tunnel positioning is crucial for successful anterior cruciate ligament reconstruction (ACLR), yet traditional arthroscopic techniques face significant challenges in spatial orientation and precise anatomical localization. This study presents a novel marker-less computer-assisted navigation system that integrates three-dimensional femoral modeling with real-time arthroscopic guidance. The system employs advanced image processing techniques for accurate condyle segmentation and implements the Bernard and Hertel (BH) grid system for standardized positioning. A curvature-based feature extraction approach precisely identifies the capsular line reference (CLR) on the lateral condyle surface, forming the foundation for establishing the BH reference grid. The system’s two-stage registration framework, combining SIFT-ICP algorithms, achieves accurate alignment between preoperative models and arthroscopic views. Validation results from expert surgeons demonstrated high precision, with 71.5% of test groups achieving acceptable or excellent performance standards (mean deviation distances: 1.12–1.86 mm). Unlike existing navigation solutions, our system maintains standard surgical workflow without requiring additional surgical instruments or markers, offering an efficient and minimally invasive approach to enhance ACLR precision. This innovation bridges the gap between preoperative planning and intraoperative execution, potentially improving surgical outcomes through standardized tunnel positioning.

## 1. Introduction

Arthroscopic anterior cruciate ligament reconstruction has become the standard surgical procedure for treating ACL injuries, offering patients minimal invasiveness and faster recovery. During these procedures, surgeons operate through small incisions while viewing internal structures through an arthroscope displayed on a screen. Despite its advantages, this approach presents significant challenges in terms of hand–eye coordination and spatial orientation. Studies have shown significant challenges in achieving accurate tunnel positioning during arthroscopic ACLR procedures, even for experienced surgeons. Research indicates that surgical technique errors, particularly tunnel malposition, remain the primary reason for ACL reconstruction failure [1]. A systematic review revealed that non-anatomic tunnel placement rates can be as high as 25–30% using traditional techniques [2], with femoral tunnel malposition being particularly problematic in both primary and revision cases [3]. Recent clinical data suggest that even with conventional arthroscopic guidance, the deviation from the ideal tunnel position can range from 2 to 4 mm, highlighting the technical demands of the procedure [4].

The success of ACLR largely depends on the accurate positioning of the femoral tunnel, which directly affects the graft’s biomechanical function and the knee’s post-operative stability. Traditional arthroscopic techniques rely heavily on the surgeon’s experience and their ability to mentally reconstruct three-dimensional anatomical relationships from two-dimensional arthroscopic images. This cognitive burden, combined with the restricted field of view through the arthroscope, makes precise tunnel placement particularly challenging.

In an effort to address these challenges, various reference systems have been developed to improve surgical accuracy. Hart et al. introduced the “apex of the deep cartilage” technique for femoral tunnel placement, which provided some standardization for anatomic positioning [5]. However, there remains a considerable gap in establishing reliable references, as traditional methods like clock-face referencing have shown limited reliability and precision in anterior cruciate ligament reconstruction. Although the CLR, visible as a “white line” corresponding to the capsular attachment on the medial aspect of the lateral femoral condyle, has emerged as a potential anatomical landmark [6], its practical application in surgical navigation remains challenging despite the development of fluoroscopic-based navigation systems [7].

To overcome these limitations and enhance surgical precision, we propose an integrated navigation system that combines precise three-dimensional modeling of the lateral femoral condyle with real-time arthroscopic guidance. Our system employs advanced image processing techniques for accurate condyle segmentation and implements the Bernard and Hertel (BH) grid system [8] for standardized positioning. This approach aims to bridge the gap between preoperative planning and intraoperative execution, providing surgeons with intuitive and accurate guidance during ACLR procedures.

Recent advances in video-based arthroscopic navigation have shown promise, yet significant challenges persist. For instance, Raposo et al. developed a navigation system using fiducial markers attached to both anatomy and instruments to enhance arthroscopic tracking [9]. While their system demonstrated impressive accuracy with footprint placement errors as low as 2.5 mm, it requires additional surgical incisions for marker placement, which partially compromises the minimally invasive nature of arthroscopic procedures. Moreover, their approach necessitates the time-consuming semi-automatic segmentation of pre-operative images, which can take up to 3 h for MRI data and 30 min for CT scans. The additional steps for marker placement and removal add approximately 9 min to the procedure time. These limitations underscore the need for a marker-less navigation solution that can maintain high accuracy while preserving the minimally invasive benefits of arthroscopic surgery.

The main contributions of this work include the development of a novel marker-less computer navigation system that integrates seamlessly with existing arthroscopic workflows without requiring additional surgical instruments or markers; implementation of a real-time tracking algorithm that maintains accurate registration of the BH grid with anatomical structures throughout the procedure; creation of an intuitive visualization approach that overlays standardized anatomical reference data directly onto the surgeon’s familiar arthroscopic field of view; and validation of the system’s effectiveness through expert evaluation, demonstrating high accuracy in femoral tunnel positioning, with 71.5% of test groups achieving acceptable or excellent performance standards.

## 2. Materials and Methods

### 2.1. System Overview

**Figure 1:**
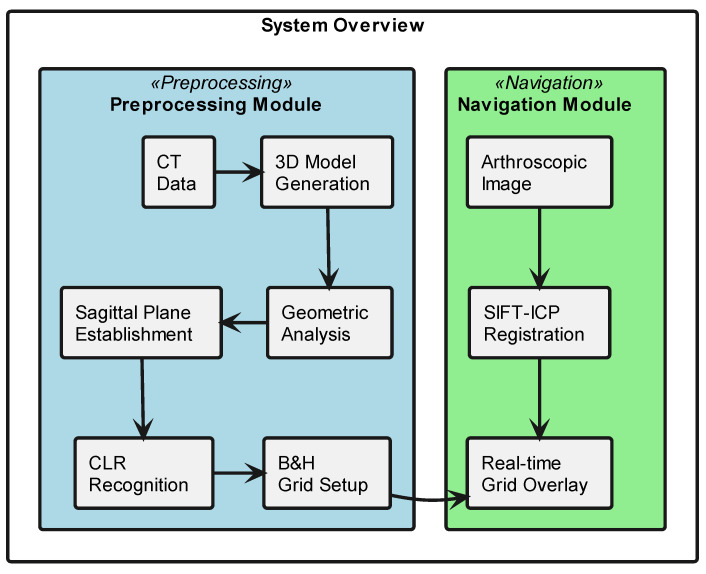
System architecture for computer-assisted ACL reconstruction navigation. This schematic diagram shows the two main components of the surgical navigation system. The preprocessing module (light blue) processes preoperative CT data to establish the surgical planning grid. The navigation module (light green) enables real-time surgical guidance through arthroscopic image registration and grid overlay. Arrows indicate data flow between components.

The proposed computer-assisted navigation system consists of two primary components: a three-dimensional femoral model analysis module and an arthroscopic navigation module, working together to provide precise guidance during ACLR procedures (as shown in Figure 1). The workflow encompasses three phases, preoperative processing, anatomical feature extraction, and real-time navigation.

In the preoperative phase, the system processes patient CT data to generate a detailed 3D model of the distal femur. This model undergoes systematic analysis, including initial sagittal plane establishment and iterative optimization of condylar profiles through elliptical fitting. A key innovation lies in the curvature-based feature extraction approach, which combines geometric analysis with anatomical constraints to precisely identify the CLR on the lateral condyle surface. This forms the foundation for establishing the BH reference grid.

The intraoperative navigation phase implements a two-stage registration framework that aligns the preoperative model with arthroscopic views. The registration utilizes a SIFT-ICP algorithm, where SIFT features provide initial alignment followed by ICP refinement for precise spatial correspondence. The system overlays the BH grid onto real-time arthroscopic feeds, providing standardized anatomical references for precise tunnel positioning.

The hardware implementation integrates with standard arthroscopic equipment through a video capture system, requiring no additional surgical instruments or tracking devices. The system maintains clinical workflow efficiency while providing enhanced visualization through semi-transparent grid overlay, enabling surgeons to accurately determine tunnel positions within their familiar arthroscopic view.

### 2.2. 3D Femoral Model Processing

**Figure 2:**
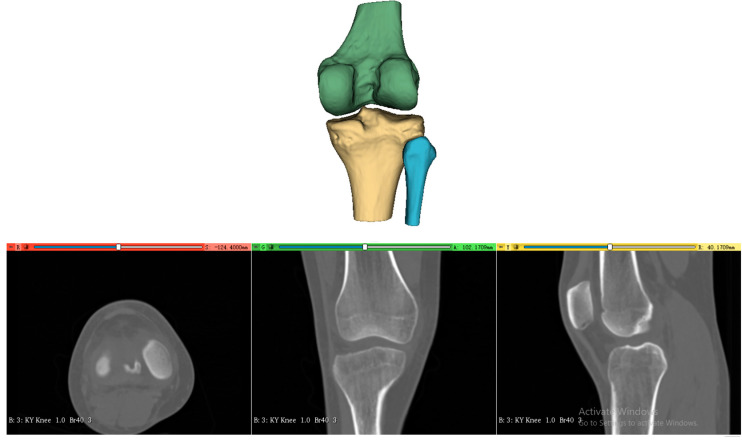
Three-dimensional surface model reconstruction of the distal femur from CT images.

The processing of the three-dimensional femoral model consists of two essential steps, reconstruction of the distal femoral surface model and establishment of the initial sagittal plane. Starting with high-resolution CT data of the patient’s knee (slice thickness 1 mm, pixel spacing 0.5 mm), the system first reconstructs the three-dimensional surface model of the distal femur, as shown in Figure 2. This reconstruction process utilizes threshold-based segmentation that takes advantage of the natural contrast between bone and surrounding tissues in CT images, followed by morphological operations to refine the initial segmentation. The resulting volumetric data are then converted into a surface mesh model through a marching cubes algorithm, producing a detailed representation of the distal femoral surface.

**Figure 3:**
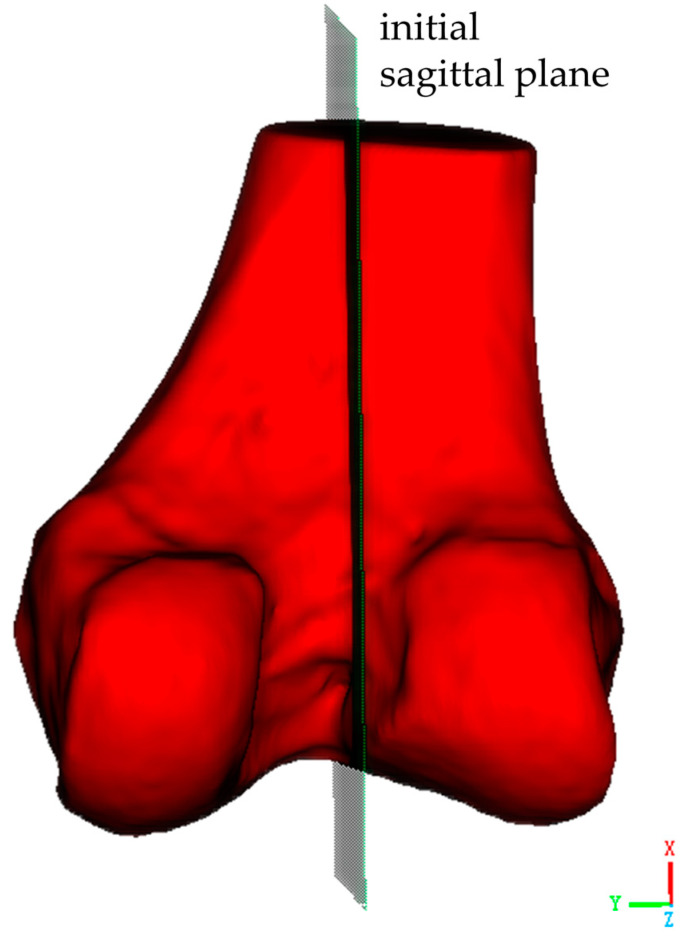
Establishment of the initial sagittal plane for the distal femoral model.

The second critical step involves establishing the initial sagittal plane of the distal femoral model. As shown in Figure 3, this begins with the identification of key anatomical landmarks at the distal femur, including the medial condyle, lateral condyle, and the intercondylar notch, which is a distinctive concave area between the condyles. Two reference points are selected along the central line of the intercondylar notch, one at its anterior margin and one at its posterior margin. These reference points are used to define a sagittal plane. This sagittal plane is a longitudinal section that is perpendicular to both the coronal and transverse planes, and it should be perpendicular to the transverse plane of the distal femur while dividing the medial and lateral condyles along the central line of the intercondylar notch.

### 2.3. Geometric Analysis and Optimization of Femoral Condylar Profiles

**Figure 4:**
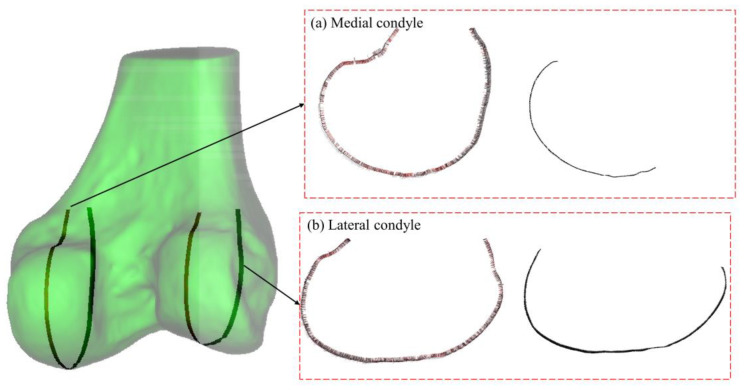
Curvature-based feature identification of femoral condyles. (**a**) Medial condyle, **left**: curvature vector distribution along sagittal contour; **right**: determined articular region. (**b**) Lateral condyle, **left**: curvature vector distribution along sagittal contour; **right**: determined articular region. Color intensity corresponds to curvature magnitude in all panels.

The geometric analysis of the distal femoral model focuses particularly on the contour characteristics of the condylar articular region in the sagittal plane, especially near the most prominent central portions. As shown in Figure 4, the sagittal contours of both medial and lateral condyles are first extracted. The sagittal plane of the femoral condyles refers to an anatomical plane passing through the anterior–posterior direction of the condyles, dividing them into medial and lateral portions. The sagittal contour represents the shape and structure of the femoral condyles observed in this plane, displayed through a longitudinal section. These contour curves are then parameterized as ($x\left(t\right)$, $y\left(t\right)$), where $x\left(t\right)$ and $y\left(t\right)$ represent the coordinates on the plane. For a parameterized curve, the curvature κ is defined as follows:

(1) $$\kappa \left(t\right)=\frac{\left|x^{\prime }\left(t\right)y^{\prime \prime }\left(t\right)-y^{\prime }\left(t\right)x^{\prime \prime }\left(t\right)\right|}{{\left({x^{\prime }\left(t\right)}^{2}+{y^{\prime }\left(t\right)}^{2}\right)}^{\frac{3}{2}}}$$

where $\left(x^{\prime }\left(t\right),y^{\prime }\left(t\right)\right)$ denotes the first derivative representing the tangent vector, and $\left(x^{\prime \prime }\left(t\right),y^{\prime \prime }\left(t\right)\right)$ represents the second derivative, indicating the rate of change of the normal vector. This curvature formula quantifies the degree of bending at each point along the curve and is used to identify characteristic points on the articular surface contour.

**Figure 5:**
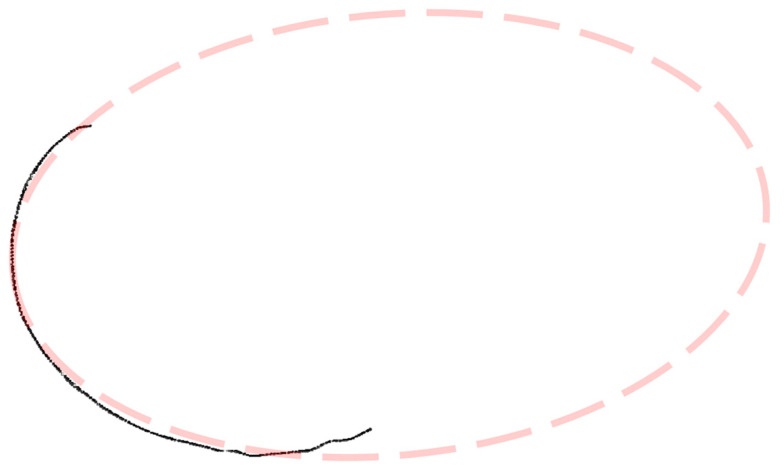
Elliptical fitting of condylar sagittal contours.

In the geometric analysis of the condylar sagittal contour curves, an elliptical fitting approach is employed for precise morphological characterization, as illustrated in Figure 5. Initially, a set of discrete data points $\left(x_{i},y_{i}\right)$ exhibiting elliptical distribution is obtained from the condylar sagittal contour curves. The following algebraic form of the general conic equation for the ellipse is used for fitting:

(2) $$Ax^{2}+Bxy+Cy^{2}+Dx+Ey+F=0$$

A design matrix X is constructed with rows in the form of $\left[x_{i}^{2},x_{i}y_{i},y_{i}^{2},x_{i},y_{i},1\right]$, and the coefficient vector $p={\left[A,B,C,D,E,F\right]}^{T}$ is solved using least squares optimization. To ensure that the fitted result maintains an elliptical shape, the constraint $B^{2}-4AC<0$ is imposed. Singular value decomposition is employed to enhance computational stability and accuracy.

**Figure 6:**
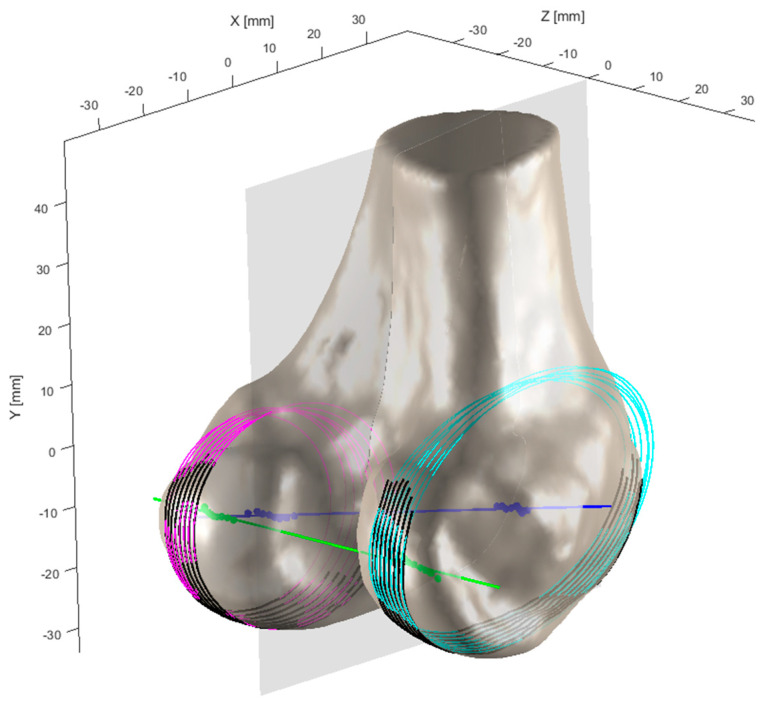
Modified sagittal contours of the femoral condyles after geometric optimization. Pink contours: These represent the parallel sectioning planes cutting through the medial condyle, creating elliptical sections. Cyan contours: These represent the parallel sectioning planes cutting through the lateral condyle, creating elliptical sections. Blue line: This represents the epicondylar axis, which runs between the medial and lateral epicondylar points. Green lines: These represent reference lines used in the iterative adjustment process to determine the optimal cutting plane orientation. Gray semi-transparent plane: This represents the sagittal cutting plane that is being iteratively adjusted to achieve perpendicularity with the epicondylar axis.

Following Fischer et al.’s methodology for femoral axis identification [10,11] and as shown in Figure 6, the adjustment of the sagittal plane orientation involves cutting both medial and lateral condyles with a series of parallel sections. When the cutting plane is not perpendicular to the epicondylar axis, the elliptical sections exhibit eccentricity. When these ellipses are superimposed or projected onto a common plane parallel to the section, their centroids or foci appear dispersed. As the cutting plane approaches perpendicularity, the focal dispersion decreases, and the sectional eccentricity weakens, with foci completely coinciding at perfect perpendicularity. The epicondylar axis, defined by the line between the medial and lateral epicondylar points, is approximately parallel to the distal femoral articular surface and is considered perpendicular to the femoral mechanical axis.

**Figure 7:**
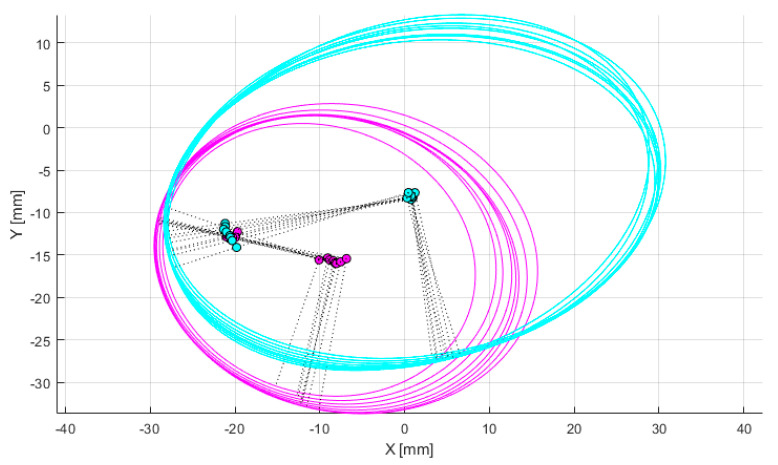
Optimization of femoral condylar sagittal profiles based on eccentricity and dispersion minimization criteria. Cyan curves: Represent the elliptical contours of the lateral condyle, generated through parallel sectioning planes. Pink curves: Represent the elliptical contours of the medial condyle, also generated through parallel sectioning planes. Gray dotted lines: Connecting lines between elliptical foci, used for evaluating condylar geometry. Green points: Indicate positions of elliptical foci. Purple points: Represent specific anatomical reference points on the medial condyle.

Through iterative adjustment of the plane orientation, as demonstrated in Figure 7, the optimal cutting plane perpendicular to the epicondylar axis is determined. The process begins with an initial orientation and defines convergence thresholds and maximum iterations. The medial and lateral condyles are sectioned, and the eccentricity and focal dispersion of the elliptical sections are calculated. In each iteration, the orientation is fine-tuned, and these metrics are recalculated. The direction is updated if the new metrics show improvement, and the iteration stops when either the metric changes fall below the threshold or the maximum iteration count is reached. This iterative algorithm systematically adjusts the cutting plane through a series of sections to approach the most prominent portions of both femoral condyles, aiming to minimize eccentricity. The complete process of optimization of femoral condylar sagittal profiles can be found in Video S1 of the Supplementary Materials.

**Figure 8:**
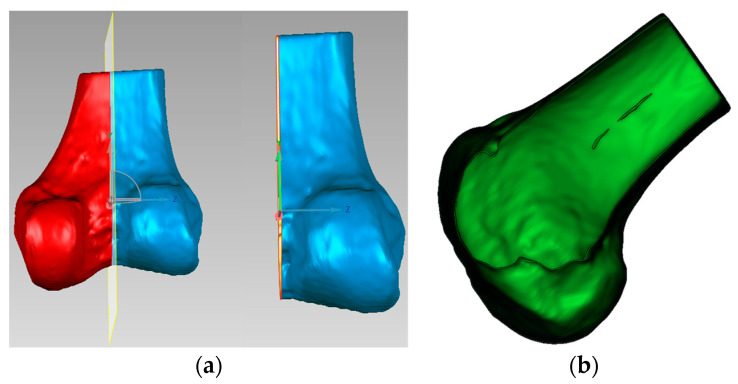
Distal femoral resection strategy and preserved condylar geometry: (**a**) implementation of modified sagittal plane for distal femoral resection; (**b**) resulting preserved lateral condylar portion after virtual resection planning.

Finally, as shown in Figure 8, the modified sagittal plane is used to cut the distal femoral model, removing the medial condylar region while preserving the lateral portion. The lateral condyle holds significant anatomical and surgical importance in ACL reconstruction surgery. It provides crucial anatomical landmarks for determining the correct position of the femoral tunnel, which is essential for restoring normal knee joint mechanical function. The morphology and position of the lateral condyle offer anatomical references for surgeons to evaluate and ensure proper graft placement, thereby improving post-operative knee stability and preventing graft loosening or displacement. Moreover, precise utilization of the lateral condyle for tunnel drilling and graft fixation can reduce the risk of perioperative tissue damage and post-operative complications. Ultimately, surgeons can adapt their surgical techniques based on the specific anatomical characteristics of the patient’s lateral condyle, accommodating individual variations to improve surgical success rates and patient recovery outcomes.

### 2.4. Extraction and Analysis of Lateral Condyle Feature Curves

The extraction of lateral condyle feature curves represents a crucial step for achieving precise navigation. This solution adopts a multi-step curve extraction strategy based on anatomical constraints, combining geometric feature analysis and anatomical knowledge to ensure the accuracy and reliability of the extraction results.

#### 2.4.1. Curvature-Based Feature Curve Extraction on Lateral Condyle Surface

**Figure 9:**
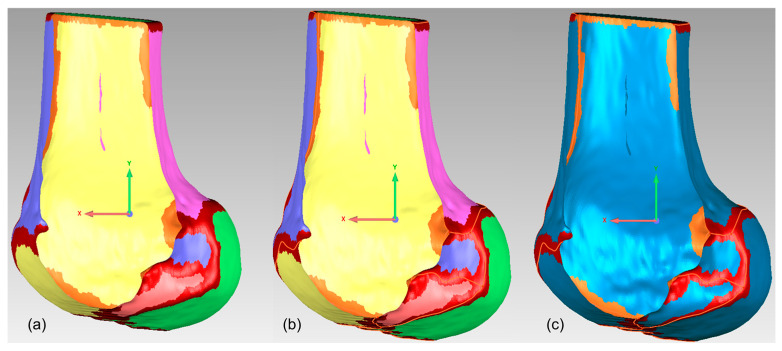
Feature curve extraction on 3D mesh based on principal curvature analysis: (**a**) results of curvature computation; (**b**) feature point identification in high-curvature regions; and (**c**) connection of feature points into continuous curves.

For the obtained 3D model of the lateral condyle, curvature analysis is first performed to identify key feature points. Given a 3D mesh model $M=\left(V,E,F\right)$, we employ discrete differential geometry methods to calculate local curvature characteristics for each vertex $v_{i}\in V$ on the mesh. The process begins with constructing a local coordinate system for vertex $v_{i}$ and considering its one-ring neighborhood $N\left(v_{i}\right)$. By computing the mixed covariant derivative matrix and solving the eigenvalue problem, we obtain the principal curvature values $\kappa_{1}$ and $\kappa_{2}$, which are then used to calculate the Gaussian curvature $K\left(v_{i}\right)=\kappa_{1}\cdot \kappa_{2}$. Based on these curvature values, feature points in high-curvature regions are identified using a threshold τ, defined as $P_{f}=\left\{v_{i}\in V\right|\;\left|K\left(v_{i}\right)\right|>\tau \}$. For connecting these feature points into continuous curves, we implement a region growing algorithm that starts from a seed point $p_{s}$ with the highest curvature value in $P_{f}$. The algorithm iteratively searches for neighboring points that simultaneously satisfy three criteria, spatial proximity ($\left|\left|p_{i}-p_{j}\right|\right|\leq \;\varepsilon$), curvature consistency ($\left|K\left(p_{i}\right)-K\left(p_{j}\right)\right|\leq \;\sigma_{k}$), and directional continuity ($∠\left(t_{i},t_{j}\right)\leq \theta_{max}$, where $t_{i}$ and $t_{j}$ denote the unit tangent vectors at adjacent points). When no more qualified points can be found, a new seed point is selected from the remaining feature points. As illustrated in Figure 9, the complete workflow consists of Gaussian curvature computation, feature point identification, and curve generation through the constrained region growing process.

#### 2.4.2. Anatomical Region-Based Feature Curve Refinement

The lateral femoral condyle is located at the lateral distal part of the femur, separated from the medial condyle by the intercondylar fossa, connected to the patellar articular surface anteriorly, and it forms the posterolateral condylar surface posteriorly. The lateral condylar region is defined as follows:

(3) $$\Omega_{L}=\left\{p\in R^{3}|\langle p-c,e_{i}\rangle \in \left[l_{i},u_{i}\right],i=1,2,3\right\}$$

where c represents the anatomical center of the lateral condyle, $\left[l_{i},u_{i}\right]$ denotes the anatomical boundaries in each direction, and $e_{i}$ represents the unit vectors of the anatomical coordinate system.

**Figure 10:**
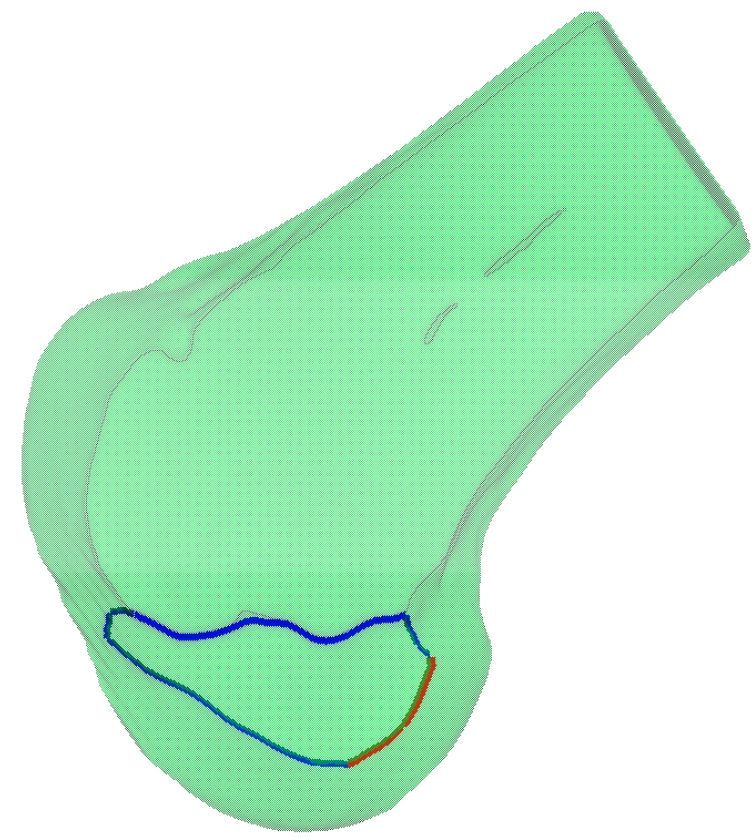
Anatomically constrained feature curve extraction on the lateral femoral condyle. The blue curve represents the curvature-based feature curve extraction, while the red curve illustrates the anatomical region-based feature curve refinement applied to the blue curve.

Based on the anatomically defined lateral condylar region $\Omega_{L}$, we perform spatial filtering on the previously extracted feature curves to obtain the clinically relevant portions. The feature curves are trimmed by intersecting them with the boundary of $\Omega_{L}$, resulting in two distinct curve segments, as shown in Figure 10. The red segment represents the clinically significant CLR, while the blue segment corresponds to the anterior articular margin. This anatomically constrained filtering ensures that the extracted geometric features accurately reflect the clinically relevant anatomical structures of the lateral femoral condyle.

### 2.5. Establishment of BH Grid on the 3D Lateral Condylar Model

**Figure 11:**
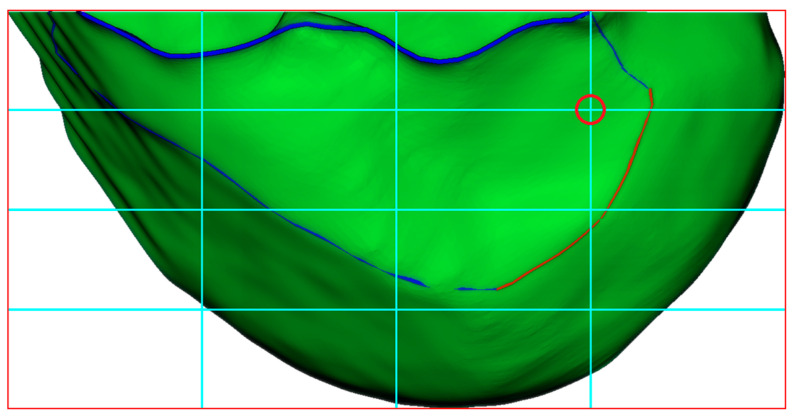
Construction of BH Grid with Reference to Blumensaat’s Line. The blue curve represents the curvature-based feature line extraction, while the red curve demonstrates the anatomical region-based feature curve refinement applied to the blue curve. The cyan lines represent the BH Grid. The red circle marks the position of the ACL femoral footprint center.

In the standard lateral position (ensuring the complete overlap of medial and lateral condyles), the Blumensaat’s line is identified by delineating a smooth continuous curve along the osseous contour of the intercondylar roof between the anterior and posterior boundary points. Let B(s) represent this curve where s ∈ [0, L] denotes the arc length parameter. Using Blumensaat’s line as the primary reference, the following two key measurement directions are established: the deep-shallow diameter line D(t), parallel to Blumensaat’s line, extending from the posterior margin to the anterior margin of the lateral condyle, where t ∈ [0, 1]; and the height line H(h), perpendicular to Blumensaat’s line, extending from the intercondylar roof to the most inferior point of the lateral condyle, where h ∈ [0, 1]. Within this coordinate system, the center of the ACL femoral footprint is located at P_ACL = P(t, h), where t = 24.8% from the posterior margin, and h = 28.5% from the roof [12]. As shown in Figure 11, the Bernard and Hertel grid is established on the lateral condyle with reference to Blumensaat’s line, where the red circle indicates the aforementioned ACL femoral footprint center. This standardized anatomical localization method provides a precise tunnel positioning reference for ACL reconstruction surgery, carrying significant clinical implications.

### 2.6. Real-Time BH Grid Overlay

**Figure 12:**
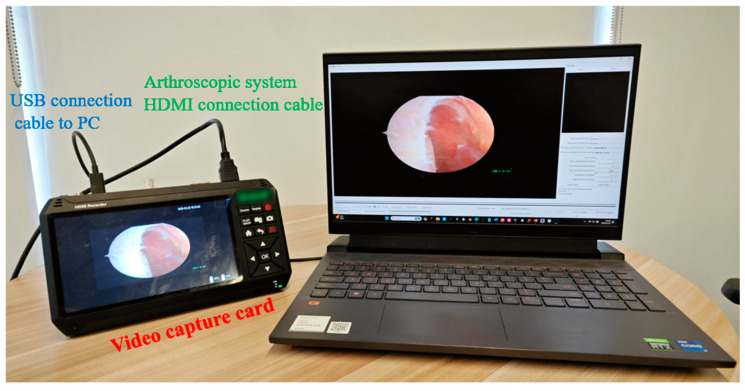
Video capture system setup for real-time arthroscopic visualization.

A video capture system is integrated with the arthroscopic equipment to enable real-time visualization and recording, as shown in Figure 12. The system comprises a compatible video capture card connected to the arthroscope’s output and computing device, configured with appropriate drivers and software to optimize image quality parameters.

The process begins with arthroscopic image enhancement using adaptive histogram equalization, followed by capsular line feature extraction through Canny edge detection (σ=1.2, $T_{low}=0.3$, $T_{high}=0.7$). Simultaneously, we reconstruct 3D lateral condylar curves from CT data, as described in Section 2.4.2. The spatial registration between arthroscopic and CT imaging modalities is formulated as a mapping function f: X → Y, where X denotes the arthroscopic image space, and Y represents the CT image space. The registration workflow employs the scale-invariant feature transform (SIFT) algorithm, where key point detection is accomplished through the Difference of Gaussian (DoG) operator [13], defined as follows:

(4) $$D\left(x,y,\sigma \right)=L\left(x,y,k\sigma \right)-L\left(x,y,\sigma \right)$$

where $L\left(x,y,\sigma \right)=G\left(x,y,\sigma \right)*\;I\left(x,y\right)$ represents the scale–space representation. Here, $I\left(x,y\right)$ is the input image, ∗ denotes the convolution operation, and k is the scale factor between adjacent scales, computed as $k=2^{\frac{1}{s}}$, where s is the number of intervals per octave. The Gaussian kernel function G is defined as follows:

(5) $$G\left(x,y,\sigma \right)=\frac{1}{\left(2\pi \sigma^{2}\right)\exp\left(-\frac{x^{2}+y^{2}}{2\sigma^{2}}\right)}$$

where σ is the standard deviation of the Gaussian distribution.

For each detected key point, SIFT generates a 128-dimensional feature descriptor vector $v=\left[v_{1},\;v_{2},\;\ldots ,\;v_{128}\right]$. Each dimension is computed through local gradient orientation histograms according to the following:

(6) $$\;v_{i}=\sum \sum m\left(x,y\right)w\left(x,y\right),\;i\;\in \;\left[1,128\right]$$

where $m\left(x,y\right)$ denotes the gradient magnitude at position $\left(x,y\right)$, and $w\left(x,y\right)$ represents the Gaussian weighting function for spatial weighting. The summation is performed over a 16×16 pixel neighborhood, with each $v_{i}$ representing one bin in the concatenated orientation histograms.

Feature correspondence is established using a k-d tree-based nearest neighbor algorithm. For a given feature point p, a match is identified when the following is true:

(7) $$\frac{\left\|p-q_{1}\right\|}{\left\|p-q_{2}\right\|}<\tau$$

where τ=0.75. Here, $\left\|\cdot \right\|$ denotes the Euclidean distance, $q_{1}$ is the nearest neighbor to p in the feature space, and $q_{2}$ is the second-nearest neighbor. This ratio test effectively filters out ambiguous matches.

In the second stage, the registration is refined through the iterative closest point (ICP) algorithm [14], which optimizes the rigid transformation matrix T by solving the following:

(8) $$T=argmin\;\sum {\left|\left|Rx_{i}+t-y_{i}\right|\right|}^{2}$$

In this expression, $R\in \mathrm{SO}\left(3\right)$ denotes the 3×3 rotation matrix, $t\in R^{3}$ represents the translation vector, and $\left(x_{i},y_{i}\right)$ are corresponding point pairs from the source and target point sets. The iteration process continues until the root mean square error (RMSE) converges to 0.8±0.2 mm, ensuring robust spatial alignment between the two imaging modalities.

**Figure 13:**
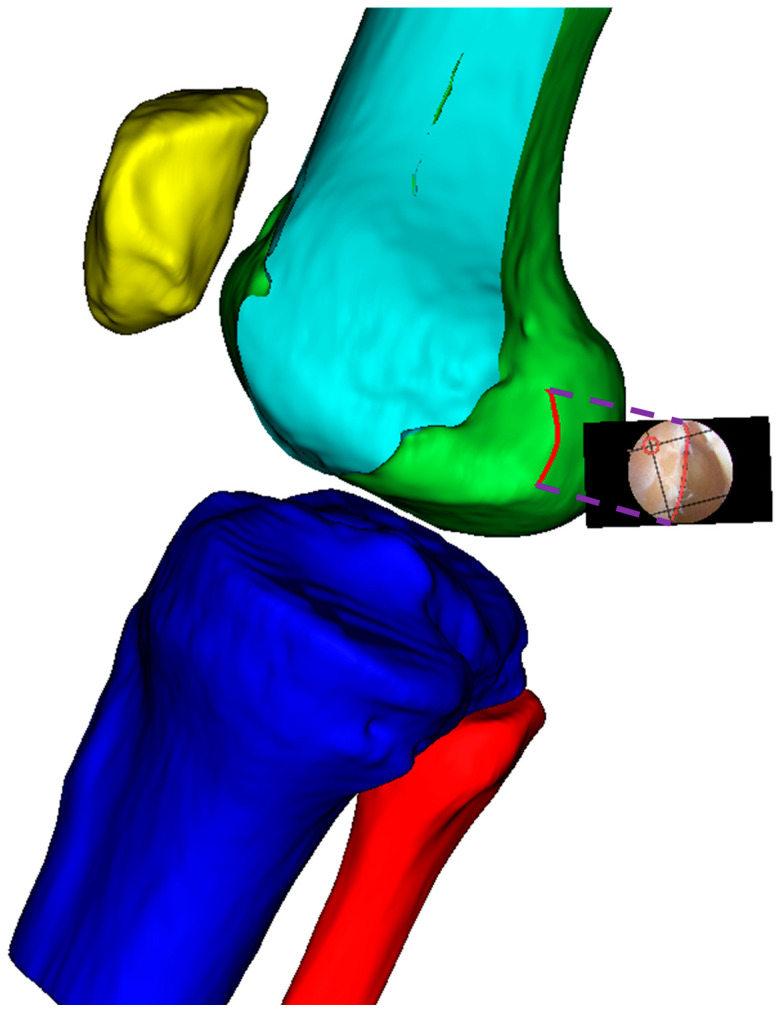
The registration of arthroscopic and preoperative CT images based on reference capsular lines. The registration process employs the SIFT-ICP framework, where SIFT features are first extracted from both imaging modalities for initial alignment, followed by ICP refinement to achieve precise spatial correspondence. The capsular lines serve as anatomical landmarks to guide and validate the registration accuracy.

This two-stage registration framework, as shown in Figure 13, combines the global feature matching capability of SIFT with the local optimization of ICP, resulting in accurate and robust registration between arthroscopic and CT images. The method is particularly effective in handling the challenging multi-modality registration scenario while maintaining computational efficiency.

## 3. Results

**Figure 14:**
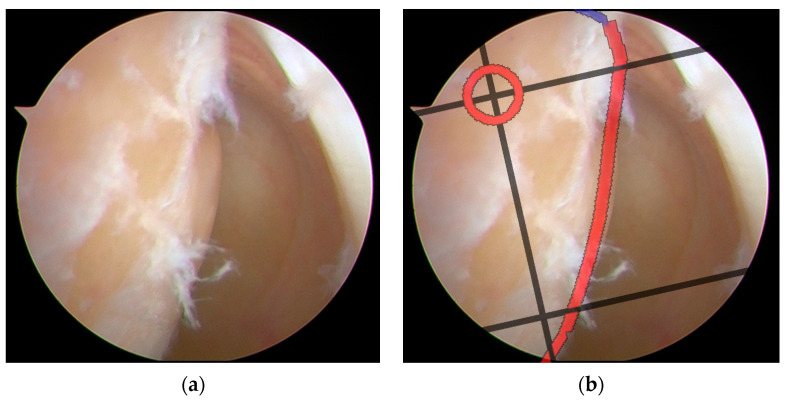
Surgical navigation for tunnel positioning in ACLR using arthroscopic images overlaid with a BH grid: (**a**) original arthroscopic image during ACLR procedure; (**b**) arthroscopic image with a superimposed BH grid for precise tunnel placement guidance. The grid system provides standardized anatomical references for optimal femoral tunnel positioning, enabling accurate and reproducible surgical navigation during ACLR procedures. The red circle marks the ideal tunnel entry point location. The red line highlights a critical anatomical boundary. The black lines form the BH Grid system, providing a standardized coordinate framework that helps surgeons navigate with precision during the ACLR procedure.

The integration of the BH grid with arthroscopic imaging for tunnel positioning demonstrated precise navigation capability during ACLR procedures. As shown in Figure 14, the system successfully superimposed a perspective-transformed and spatially calibrated 4 × 4 BH standard grid onto real-time arthroscopic video feeds.

The semi-transparent visualization of the grid maintained clear visibility of the underlying anatomical structures while providing accurate spatial references. Key anatomical landmarks, including the posterior edge of the lateral femoral condyle and Blumensaat’s line, showed precise correspondence with the grid positions. The system employed a dynamic tracking algorithm to continuously update the grid position, ensuring consistent registration with anatomical structures throughout the procedure.

The ideal tunnel entry points were prominently marked on the grid system. This intuitive visualization approach enabled surgeons to accurately determine tunnel positions within their familiar arthroscopic field of view. This enhanced visualization strategy significantly improved the precision and safety of surgical procedures by providing surgeons with standardized anatomical references directly integrated into their operative view. The system maintained stable performance under various lighting conditions and camera movements typical in arthroscopic procedures.

### Expert Concordance

**Figure 15:**
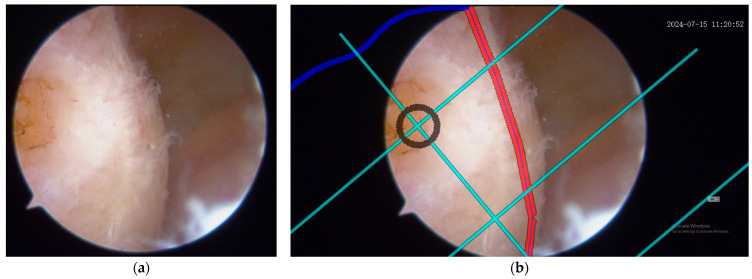
Expert validation of femoral tunnel positioning using arthroscopic RF marking and grid overlay: (**a**) representative arthroscopic images showing the femoral condyle with an experienced surgeon’s reference point marked by an RF ablation device based on conventional surgical expertise; (**b**) the same arthroscopic views with a superimposed BH grid overlay for precise tunnel placement guidance, demonstrating the spatial correlation between the surgeon-marked RF point and the grid-recommended optimal position. The black circle marks the ideal tunnel entry point location. The red line highlights a critical anatomical boundary. The cyan lines represent the BH Grid.

**Figure 16:**
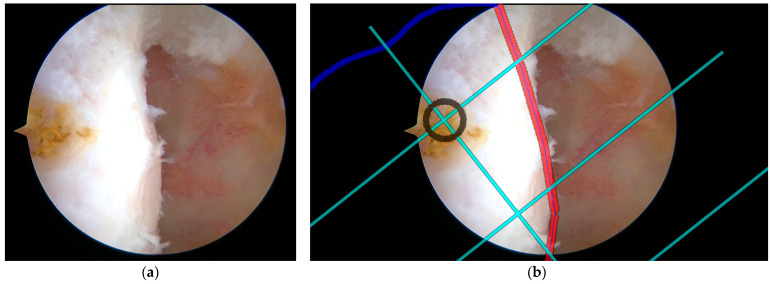
Second case of expert validation using RF marking and grid overlay: (**a**) surgeon’s RF reference point on femoral condyle; (**b**) Same view with a BH grid overlay showing spatial correlation. The black circle marks the ideal tunnel entry point location. The red line highlights a critical anatomical boundary. The cyan lines represent the BH Grid.

To validate the tunnel positioning accuracy, an experienced sports medicine surgeon first marked a reference point on the femoral condyle using a radiofrequency ablation device based on conventional surgical experience. This surgeon-determined position was then compared with the system-recommended tunnel location to assess the consistency between expert clinical judgment and computer-assisted navigation guidance. The spatial correlation between the radiofrequency (RF) marked point and the navigation-suggested optimal position served as a quantitative measure of the system’s clinical reliability in tunnel placement recommendations. As illustrated in Figure 15 and Figure 16, two representative cases demonstrating this validation methodology are presented, where the surgeon’s empirical RF marking points were compared with the navigation system’s guidance overlay to assess the concordance between expert judgment and computer-assisted recommendations.

**Figure 17:**
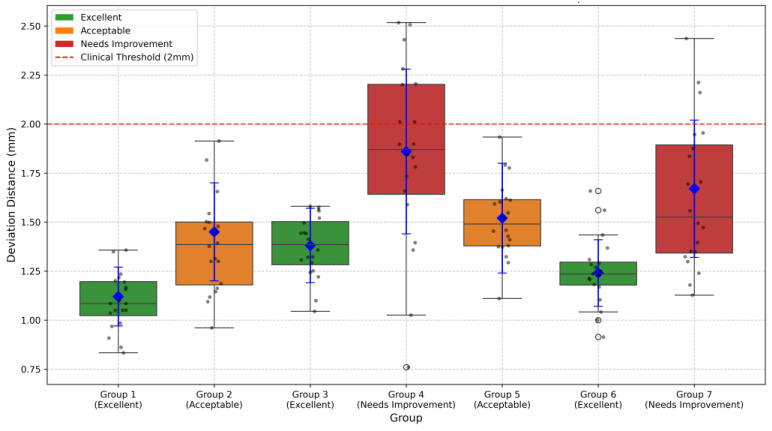
Deviation distance measurements across different groups. Boxplots show the distribution of deviation distances from the grid center (0, 0) for seven expert groups. Blue diamonds represent mean values with error bars indicating standard deviation. The red dashed line represents the 2 mm clinical threshold. The following box colors indicate quality classification: green (Excellent), orange (Acceptable), and red (Needs Improvement). Groups 1, 3, and 6 demonstrated excellent performance with mean deviation distances of 1.12 ± 0.15 mm, 1.38 ± 0.19 mm, and 1.24 ± 0.17 mm, respectively. Groups 2 and 5 showed acceptable performance (1.45 ± 0.25 mm and 1.52 ± 0.28 mm), while Groups 4 and 7 needed improvement with higher deviation distances (1.86 ± 0.42 mm and 1.67 ± 0.35 mm).

Expert concordance was evaluated using a coordinate system centered on the grid center (0, 0), analyzing RF marking points from seven groups. Based on three core metrics (mean deviation distance, coefficient of variation, and percentage within 2 mm range), the seven expert groups were classified into three categories as shown in Figure 17. Three groups (Groups 1, 3, and 6, accounting for 42.9%) achieved excellent performance with CV values below 15% and within-2 mm percentages exceeding 90%. Two groups (Groups 2 and 5, representing 28.6%) showed acceptable results with CV values between 15 and 20% and within-2 mm percentages between 80 and 90%. The remaining two groups (Groups 4 and 7, 28.6%) needed improvement, demonstrating higher CV values (>20%) and lower within-2 mm percentages (<80%).

It is important to note that the quality metrics shown in Figure 17 only represent the concordance between expert marking and navigation system recommendations, rather than absolute accuracy of tunnel placement. The navigation system’s recommended positions are based on the gold standard established by Bernard et al. [8]. Therefore, while Groups 4 and 7 showed lower concordance with the navigation system as evidenced by their greater deviation distances, this may not necessarily indicate inferior surgical technique, but rather a deliberate deviation from the radiographically determined optimal position. The boxplot visualization clearly demonstrates the variability in expert approaches, with Groups 1, 3, and 6 showing the highest consistency with the navigation system’s recommendations. The navigation system’s recommendations are grounded in evidence-based anatomical measurements, where Bernard et al. [8] found that using the Blumensaat line (intercondylar roof line) as a reference landmark on standard lateral radiographs of the femur provides reliable anatomical guidelines for tunnel positioning.

## 4. Discussion

The proposed computer-assisted navigation system demonstrates significant potential in enhancing the precision of femoral tunnel placement during ACLR procedures. The integration of three-dimensional modeling with real-time arthroscopic guidance represents a novel approach to addressing the longstanding challenges of tunnel positioning accuracy. Our validation results, showing that 71.5% of groups achieved acceptable or excellent performance standards, suggest that the system provides reliable assistance in surgical decision-making. The mean deviation distances ranging from 1.12 to 1.86 mm indicate a high level of precision that aligns with clinical requirements for successful ACLR outcomes.

When compared to existing commercial navigation systems for ACLR, our approach offers several distinct advantages. Current commercial solutions, such as the Brainlab Knee3 and Stryker’s OrthoMap Precision Knee Navigation, typically require additional hardware components, including optical trackers or electromagnetic sensors that must be attached to surgical instruments and patient anatomy. These systems, while effective, introduce additional procedural complexity and extend operative time by approximately 15–20 min.

Recent video-based navigation research approaches, such as the system developed by Raposo et al., use fiducial markers attached to both anatomy and instruments to enhance arthroscopic tracking [9]. While their system demonstrated impressive accuracy with footprint placement errors as low as 2.5 mm, it requires additional surgical incisions for marker placement, which partially compromises the minimally invasive nature of arthroscopic procedures. Moreover, their approach necessitates time-consuming semi-automatic segmentation of pre-operative images, which can take up to 3 h for MRI data and 30 min for CT scans, with the additional steps for marker placement and removal adding approximately 9 min to the procedure time. In contrast, our marker-less approach maintains standard surgical workflow while providing enhanced visualization through grid overlay without additional instrumentation.

The system’s ability to maintain standard surgical workflow while providing enhanced visualization through grid overlay represents a significant advantage over existing navigation solutions. By eliminating the need for additional surgical instruments or tracking devices, the system preserves operational efficiency while improving spatial orientation and anatomical reference visualization. The curvature-based feature extraction method for identifying anatomical landmarks, particularly the CLR, demonstrates robust performance in establishing reliable reference points for tunnel positioning.

However, several limitations should be acknowledged. The system’s performance depends heavily on the quality of preoperative CT images and intraoperative arthroscopic visualization. While our validation focused on the concordance between expert marking and system recommendations, long-term clinical outcomes and follow-up studies are needed to definitively establish the system’s impact on surgical success rates. Furthermore, the current validation sample size, though providing valuable initial insights, would benefit from larger-scale clinical trials to establish broader applicability across varying patient populations and surgical scenarios.

## 5. Conclusions

This study presents a novel computer-assisted navigation system that successfully integrates three-dimensional femoral modeling with real-time arthroscopic guidance for ACLR procedures. The system demonstrates reliable performance in providing precise tunnel positioning guidance, with validation results showing that over 70% of expert users achieved acceptable or superior accuracy levels. The innovative approach to anatomical feature extraction and grid overlay visualization offers surgeons an intuitive and accurate tool for improving surgical precision while maintaining familiar workflow patterns.

The successful implementation of the BH grid system in real-time arthroscopic visualization represents a significant advancement in surgical navigation technology. This achievement bridges the gap between preoperative planning and intraoperative execution, potentially reducing the cognitive burden on surgeons while enhancing the consistency of tunnel placement.

Looking toward future developments, we recognize that implementing this system as a clinical decision support system requires addressing critical constraints related to security and service availability in clinical operational scenarios. Service continuity could be enhanced by adopting bio-inspired approaches similar to those proposed by Conti et al. [15], where self-organizing systems with non-fixed structures can adaptively modify their organization in response to internal and external environment changes. In service-oriented networks (SONs), nodes communicate through stimulation or suppression chains, creating emergent behaviors that defend against attacks, malfunctions, and foreign invaders. Such bio-inspired architectures could significantly improve the robustness of our navigation system, ensuring the continuous availability of services, which is a mandatory requirement in clinical settings.

The integration of FPGA technology, as demonstrated in the referenced work, could further enhance computational performance for real-time processing while maintaining system reliability. This approach would be particularly valuable for handling the intensive image processing requirements of our navigation system in clinical environments where service interruptions cannot be tolerated.

Future work should focus on expanding the system’s capabilities through larger clinical trials, the refinement of image processing algorithms, and potential adaptation to other arthroscopic procedures, while incorporating robust service continuity mechanisms inspired by biological networks. The promising results of this study suggest that computer-assisted navigation systems with enhanced service availability could become an integral part of modern arthroscopic surgery, contributing to improved surgical outcomes and standardized procedural approaches.

## Data Availability

The original contributions presented in this study are included in the article/Supplementary Material. Further inquiries can be directed to the corresponding author.

## Supplementary Materials

The following supporting information can be downloaded at: https://www.mdpi.com/article/10.3390/bioengineering12050464/s1, Video S1: Video summary.

## Abbreviations

The following abbreviations are used in this manuscript:

ACLR	Anterior Cruciate Ligament Reconstruction
BH	Bernard and Hertel
CLR	Capsular Line Reference
SIFT	Scale-Invariant Feature Transform
DoG	Difference of Gaussian
ICP	Iterative Closest Point
RMSE	Root Mean Square Error
CV	Coefficient of Variation
